# Fuel for Life: Domestic Cooking Fuels and Women’s Health in Rural China

**DOI:** 10.3390/ijerph13080810

**Published:** 2016-08-10

**Authors:** Peng Nie, Alfonso Sousa-Poza, Jianhong Xue

**Affiliations:** 1 Institute for Health Care & Public Management, University of Hohenheim, Stuttgart 70599, Germany; alfonso.sousa-poza@uni-hohenheim.de; 2 Department of Agricultural Economics, College of Economics and Management, Northwest A&F University, 3 Taicheng Road, Yangling 712100, Shaanxi, China; xjh621002@163.com

**Keywords:** household cooking fuels, health, women, rural China

## Abstract

*Background*: There is evidence that household air pollution is associated with poor health in China, and that this form of air pollution may even be more of a health concern in China than the much-publicized outdoor air pollution. However, there is little empirical evidence on the relationship between household air pollution and health in China based on nationally representative and longitudinal data. This study examines the association between the type of domestic cooking fuel and the health of women aged ≥16 in rural China. *Methods*: Using longitudinal and biomarker data from the China Family Panel Studies (*n* = 12,901) and the China Health and Nutrition Survey (*n* = 15,539), we investigate the impact of three major domestic cooking fuels (wood/straw, coal, liquefied petroleum gas (LPG)) on health status using both cross-sectional and panel approaches. *Results*: Compared to women whose households cook with dirty fuels like wood/straw, women whose households cook with cleaner fuels like LPG have a significantly lower probability of chronic or acute diseases and are more likely to report better health. Cooking with domestic coal instead of wood or straw is also associated with elevated levels of having certain risks (such as systolic blood pressure) related to cardiovascular diseases. *Conclusions*: Our study provides evidence that using cleaner fuels like LPG is associated with better health among women in rural China, suggesting that the shift from dirty fuels to cleaner choices may be associated with improved health outcomes.

## 1. Introduction

Globally, 40% of the population relies on solid fuels, including coal and biomass (e.g., wood, charcoal, agricultural residues and dung), for domestic cooking, thereby breathing in a large amount of particulate matter (PM) and pollutants that can be detrimental to health [1]. According to the 2010 Global Burden of Disease/Comparative Risk Assessment Project, for example, exposure to household air pollution (HAP) from cooking with solid fuels resulted in 3.5 million premature death and various other health problems (e.g., lung cancer) in 2010 [2,3]. Yet despite unprecedented economic development in China, most rural Chinese still cook with solid fuels, 47.6% with biomass and 13.5% with coal in 2011 [4], and the use of solid fuels is likely to continue in the future [5]. As a result, HAP associated with solid fuels is estimated to be the largest single environmental risk contributor and ranks sixth among all risk factors examined for bad health in China [6], resulting in over half a million premature deaths annually [7]. At the same time, although a relatively large body of epidemiological and environmental literature examines the effects of HAP on health in parts of China [6,8,9,10,11,12,13,14,15], no studies assess this risk using nationally representative data and a longitudinal setting. Consequently, the evidence on HAP and health in China as a whole is scant, often narrowly focused (i.e., on certain provinces or cities) and usually based on cross-sectional analyses with limited generalizability and little ability to address confounders. 

To address these shortcomings, this paper investigates the impacts of domestic cooking fuels on the health outcomes of rural women aged ≥16 using 2010–2012 data from the China Family Panel Studies (CFPS) and 1991–2009 data from the China Health and Nutrition Survey (CHNS). Specifically, it analyses the different health impacts associated with three major household cooking fuels: wood/straw, coal and liquefied petroleum gas (LPG). The research focuses on women in rural China because they are predominantly responsible for cooking and thus bear the brunt of the illness burdens associated with HAP [6]. 

The study contributes to the existing literature in three ways: First, it provides a comprehensive analysis of the health impacts related to domestic cooking fuels by using the most recent nationally and regionally representative datasets (the CFPS and CHNS, respectively). Second, it assesses the impact of household cooking fuels on health within a panel setting capable of capturing time-invariant individual heterogeneity. Third, by using biomarker data from the 2009 CHNS, it more closely examines the association between HAP and certain risk predictors of cardiovascular disease, evidence for which has been primarily limited by a lack of reliable biomarker data [16]. This association can shed new light on the mechanisms by which HAP from household cooking fuels may influence health outcomes. 

## 2. Prior Studies

### 2.1. Fuel-Based HAP and Respiratory Diseases

In the rapidly growing body of epidemiologic and environmental science literature on the association between fuel-based HAP and health in China, most studies present strong evidence that exposure to HAP is significantly related to an increased risk of bad health. With regard to the impact of fuel-based HAP on adult respiratory diseases, Liu et al. [17], using survey data for rural Yunyan in Guangdong Province, show that cooking with biomass fuel, as compared with LPG, increases the probability of chronic obstructive pulmonary disease (COPD) in non-smoking women aged ≥40. Similarly, using cross-sectional data for rural China (Shaanxi, Hubei and Zhejiang), Peabody et al. [13] find that, compared with other types of fuel, coal fuel is associated with an increased level of exhaled CO, an increased history of overall respiratory disease (including asthma, chronic bronchitis, emphysema, COPD and tuberculosis) and decreased forced vital capacity (FVC) among adults aged ≥18. Pan et al. [18] also show that HAP from fuel combustion for cooking and heating has adverse effects on asthma attacks among respondents aged 15–65. These findings are confirmed by a recent meta-analysis of 33 international studies on HAP’s detrimental respiratory impacts among those aged ≥65 [19], which points to a generally significant association between HAP exposure and various short-term and long-term respiratory diseases, including wheezing, breathlessness, cough, phlegm, asthma and COPD. In contrast, Xu et al. [20] find no association between domestic cooking fuels and COPD in individuals aged ≥35 in Nanjing City, while Kan et al.’s [11] matched case-control analysis of respondents aged ≥15 in Huaiyuan County, Anhui Province, identifies no link between cooking with solid fuel and tuberculosis in the presence of proper ventilation. This latter is supported by a recent review of 15 international studies [21], which suggests that the association between domestic use of solid fuels and tuberculosis is very low. 

Because lung cancer is a serious health problem in China [22,23], many studies focus on its association with fuel-based HAP. For example, one meta-analysis of 27 studies on air pollution and lung cancer risks in China provides evidence that the odds ratio (OR) of the lung cancer risk among women is 1.83 in households that use coal for heating and cooking [15]. Another review on the health effect of HAP from coal use in nine single-province/city/municipality studies (seven in China, one in Japan and another in the U.S., with a focus either on women or on men and women separately) finds that the OR of lung cancer risks among women is 1.17 [14]. This risk of death from lung cancer for women almost doubles (OR = 1.94) in studies that adjust for smoking behaviour and a history of chronic respiratory disease. A more recent meta-analysis of 23 studies, including 17 carried out in China, also suggests that coal use is correlated with lung cancer and that the risk is more pronounced among females [24]. Kim et al. [25], using a prospective cohort of women in urban Shanghai from 1996 to 2009, demonstrate that cooking with coal is particularly harmful when the kitchen has poor ventilation, and Barone-Adesi et al. [26], using a retrospective cohort of 37,272 individuals in Xuanwei County, Yunnan Province, show that smoky coal is related to a higher lung cancer risk than smokeless coal. Coal use is also associated with general lung function impairment [27,28,29]. For example, Zhou et al. [30], in a nine-year prospective cohort study among rural residents aged ≥40 in southern China, find a correlation between using clean fuels like biogas (rather than biomass) and better lung function, as well as a decrease in risk for COPD.

### 2.2. Fuel-Based HAP and Non-Respiratory Diseases

A small body of literature for China does examine the association between fuel-based HAP and predictors of cardiovascular diseases (CVD), especially high blood pressure. Among these studies, Baumgartner et al. [8] find that exposure to indoor biomass combustion is associated with an increase in systolic blood pressure (SBP) and diastolic blood pressure (DBP) among rural Chinese women aged >50. They find no such association, however, among younger cohorts aged 25–50. This latter finding is confirmed in Baumgartner et al. [31] for 280 non-smoking women aged ≥25 living in a rural region of north-western Yunnan province whose exposure to pyrolytic biomass combustion is strongly associated with blood pressure issues. Lee et al. [32] also find that the use of in-home solid fuel is significantly correlated with an increase in the probabilities of hypertension, coronary heart disease, and diabetes among adults aged ≥18 in the Putuo district of Shanghai.

Domestic cooking fuels are also linked to certain poisonous endemics. For instance, in their study of arsenic exposure associated with coal-burning in a small village in Guizhou Province, Shraim et al. [33] find that females have higher dimethylarsinic acid levels but lower percentages of inorganic arsenic and monomethylarsonic acid than males. Several studies also confirm that domestic coal combustion results in selenium poisoning and potential mercury poisoning [12,34,35]. Li and Ma [36] also identify a correlation between an increased incidence of CO poisoning and indoor coal burning, inadequate ventilation and/or inappropriate stove use. 

In the context of our study, three aspects of the extant literature are particularly relevant: First, the empirical results for China generally suggest that fuel-based HAP has an adverse impact on both respiratory and non-respiratory health outcomes. Yet most existing studies for China cover only certain provinces or cities [13], and no studies use nationally representative data. Second, except for Barone-Adesi et al. and Zhou et al. [26,30], who use retrospective data to evaluate the impact of domestic cooking fuels on lung cancer and COPD, respectively, nearly all extant studies use cross-sectional data. Third, only a limited number of studies examine the association between fuel-based HAP and CVD in China. Hence, despite recent findings on the association between HAP and blood pressure [8,31], relatively little is known about how HAP exposure from domestic cooking fuels is related to other risk predictors of CVD such as inflammation. Furthermore, as emphasized by Baumgartner et al. [8] and Baumgartner et al. [31], the evidence on the association between blood pressure (BP) and domestic cooking fuels is exclusively based on cross-sectional data. A major contribution of our study, therefore, is the provision of nationally representative results based on panel data that allow us to control for unobserved heterogeneity. Given that high blood pressure results in around half of all CVD [37], another contribution is our assessment of how domestic cooking fuels affect some major risk predictors of CVD (such as high blood pressure), which may shed light on the mechanisms through which HAP operates on health outcomes.

## 3. Data and Methods 

### 3.1. Study Population

The China Family Panel Studies (CFPS), conducted by Peking University’s Institute of Social Science Survey, currently comprises two waves, 2010 and 2012, which cover 25 provinces/municipalities/autonomous regions representing 95% of the Chinese population. The CFPS is a nationally representative dataset and aims to capture socio-economic development, as well as economic and non-economic well-being in Chinese households [38]. The CFPS sample for our study, taken from both waves, includes 12,901 rural women aged ≥16. 

The China Health and Nutrition Survey (CHNS), administered in nine waves—1989, 1991, 1993, 1997, 2000, 2004, 2004, 2009 and 2011—covers nine provinces (Liaoning, Heilongjiang, Jiangsu, Shandong, Henan, Hubei, Hunan, Guangxi and Guizhou). The CHNS allows us to capture the spatio-temporal dynamics of the social, economic and health situations of the Chinese population [39]. Our CHNS sample, taken from the 1991 to 2009 waves, includes 15,539 rural women aged ≥16.

Regarding the aim of our study, two key points are worth mentioning: First, the advantage of the CFPS is that it is the first dataset that contains nationally representative information on health and cooking fuels. Second, the CHNS, although not nationally representative, has three other advantages that we exploit in the analysis: a long-running panel, information on time exposure to cooking and, particularly important, biomarker information that allows a unique analysis of the association between cooking fuels and certain risk factors of CVDs.

### 3.2. Health Measures

The CFPS captures illness or disease with the following question: “Have you felt any physical discomfort during the preceding two weeks”, which is coded 1 if the answer is yes, and 0 otherwise. Similarly, the CHNS captures general health as follows: “Have you suffered from a chronic or acute disease during the past 4 weeks?”, which is represented by a dummy coded 1 if the answer is yes, and 0 otherwise. The CFPS also assesses self-reported health (SRH) status on a 5-point scale (1 = excellent, 2 = very good, 3 = good, 4 = fair and 5 = poor). We rescale these values from 1 = poor to 5 = excellent to bring them into line with the corresponding 4-point CHNS item (1 = bad; 2 = fair; 3 = good; 4 = excellent). It should be noted that SRH covers not only mental and physical health but also subjective experience of acute and chronic diseases and overall feelings of well-being [40].

For the risk predictors of CVD, we use CHNS data on systolic and diastolic blood pressure (SBP/DBP) and inflammation as proxies. In the CHNS, blood pressure information is based on three successive pairs of blood pressure measurements taken by a health professional using a mercury sphygmomanometer at intervals of at least one minute. We calculate the average values of SBP and DBP based on the second and third measurements to overcome potential measurement biases [41]. We follow the conventional way of dealing with C-reactive protein (CRP) values and remove those with values beyond 10 mg/dL [42]. Inflammation is only available in the 2009 CHNS wave and it captured by a dummy variable equal to 1 if CRP ≥ 3 mg/dL and 0 otherwise [43] mainly because values above 3 mg/dL are associated with high risk of future cardiovascular events [42]. 

We also test for several other major disease symptoms potentially related to fuel-based HAP using binary dummies. In the CFPS data, these are fever, pain, cough and palpitation, by asking each respondent based on the question: “What type of discomfort have you felt during the past 2 weeks?” In the CHNS, they are fever, cough, asthma, eye disease and heart disease/chest pain, based on the question: “Did you have any of those symptoms during the past 4 weeks?”.

### 3.3. Household Cooking Fuels (HCF)

As in Peabody et al. [13], we investigate three major household cooking fuels—wood/straw, coal and liquefied petroleum gas (LPG)—which account for 84% of total household fuel consumption in the CFPS and 88% in the CHNS. The household cooking fuel variable can thus take three values: 0 = wood/straw, 1 = coal and 2 = LPG, with wood/straw as the reference group. As noted by Peabody et al. [13], fuel types might be better proxies for pollution exposure than the type of stove used in the household, although both are only indirect measures of exposure to HAP. The fuel-based approach is, however, regarded as the most accurate technique for assessing HAP in developing countries [44]. 

### 3.4. Independent Variables

Our independent variables encompass three subgroups: individual, family and community characteristics (for a detailed description of all independent variable definitions, see appendix Table A1). Individual controls are age, educational level, marital status, job status and current smoking behaviour. The definitions of the individual variables are the same for both the CFPS and the CHNS except for time spent cooking (hours/day), which is only available in the CHNS. The family controls are household size and household income, which for ease of comparability is adjusted to 2012 in the CFPS and to 2011 in the CHNS. In addition, we also introduce the availability of safe drinking water, existence of flushing toilets, clean trash treatment (for the CFPS), existence of excreta around the living place (for the CHNS) and availability of electricity. For the community characteristics, in the CFPS and the CHNS, we employ the distance (in km) in the community to the nearest health facilities. In the CHNS, we also introduce the location of health facilities in the community.

### 3.5. Estimation Approaches

To investigate the association between domestic cooking fuels and general disease (or specific symptoms) in a cross-sectional setting in the CFPS and the CHNS, we adopt a probit regression model of the following form:

(1) $$GD_{i}=\alpha_{0}+\alpha_{1}HCF+\alpha_{2}I_{i}+\alpha_{3}F+\alpha_{4}C+\alpha_{5}Y+\alpha_{6}P+\varepsilon_{i}$$

where $GD_{i}$ is a binary variable indicating general chronic or acute disease or a specific disease symptom of individual *i*, and *HCF* denotes dummies for the three household cooking fuels, wood/straw, coal and liquefied petroleum gas, with wood/straw as the reference group (applied to both the CFPS and the CHNS). $I_{i}$ is a vector of individual *i*’s characteristics, *F* is a vector of family characteristics and *C* is a vector of community characteristics. *Y* is a vector of year dummies (with 2010 and 1991 as the reference year in the CFPS and CHNS, respectively), and *P* is a vector of provincial dummy variables (with Liaoning as the reference province in both datasets). $\alpha_{1}$ is the key coefficient of interest, and $\varepsilon_{i}$ is the error term. 

To assess the effects of household cooking fuels on certain risk predictors of cardiovascular diseases, we use an ordinary least squares estimation (OLS) for SBP and DBP, and a probit estimation for inflammation with the specifications similar to Equation (1). For SRH, we estimate the impact of household cooking fuels with an ordered probit model using data from the CFPS and the CHNS and specifications similar to Equation (1). 

As regards the association between domestic cooking fuels and disease in general (or specific symptoms), since domestic cooking fuels in a household would not change within a short-term period, we account for the potential biases associated with individual unobservables by estimating the following random effects probit model with data from the CFPS and the CHNS:

(2) $$GD_{it}=I\left(X_{it}^{'}\beta +F_{t}+\alpha_{i}+\varepsilon_{it}\geq 0\right)$$

where $GD_{it}$ denotes general chronic or acute disease of individual *i* at time *t*, *I(.)* is an indicator function that equals 1 if its argument is true (0 otherwise), and $X_{it}$ is a vector of individual characteristics. $F_{t}$ captures the family characteristics, while $\alpha_{i}$ is assumed to be a random variable. 

Because SRH is ordinal in both data sets under consideration, for this variable we adopt a random effects ordered probit estimation that uses a specification similar to Equation (2):

(3) $$SRH_{it}^{*}=X_{it}\beta +\alpha_{i}+\varepsilon_{it}$$

(4) $$SRH_{it}=\{\begin{array}{c} 1,\;if\;SRH_{it}^{*}\leq c_{1}\\ 2,\;if\;c_{1}<SRH_{it}^{*}\leq c_{2}\\ \vdots \\ K,\;if\;c_{K-1}<SRH_{it}^{*} \end{array}$$

where $SRH_{it}^{*}$ (linked to the observed ordinal response categories $SRH_{it}$ is a latent variable of SRH for individual *i* at time *t*. $X_{it}$ represents observed characteristics, $\alpha_{i}$ is a random variable, *c* is a set of cut-off points $c_{1}$, $c_{2}\;$…, $c_{K-1}$ and *K* is the number of possible outcomes. 

We use a balanced panel in the CFPS and an unbalanced panel in the CHNS. In order to test for possible attrition bias in the CHNS, we employ the variable addition test proposed by Verbeek and Nijman [45], which is evaluated on the significance of an added variable (defined as the number of surveyed years that each respondent is present in the survey), and its insignificance denotes the nonexistence of attrition bias. All the health measures, together with the data and methods used, are summarized in Table A2. In order to take account of individual unobserved heterogeneity, we also estimate a corresponding panel model using data from the CHNS.

## 4. Results

### 4.1. Descriptive Statistics

**Figure A1:**
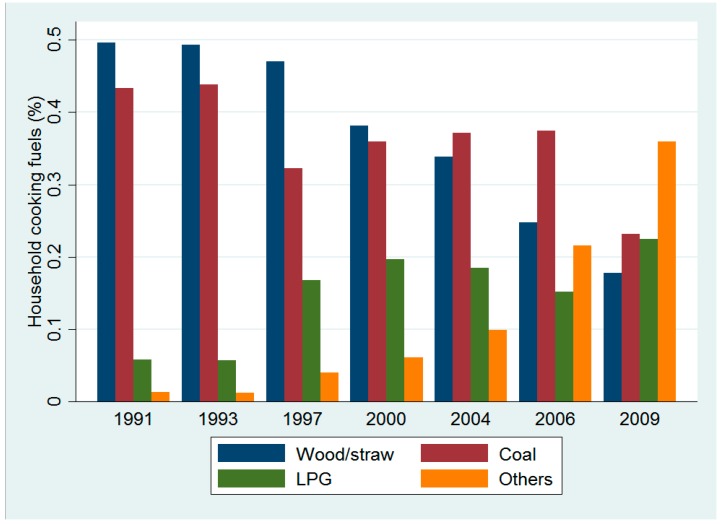
Temporal change of domestic cooking fuels from 1991 to 2009, China Health and Nutrition Survey. Note: LPG denotes liquefied petroleum gas and others represent electricity, kerosene, natural gas, charcoal and other unspecified fuels for cooking.

As appendix Table A1 shows, the average age of rural women in the CFPS is around 46, which is quite similar to the 45 years in the CHNS (see Table 2). Wood/straw is more predominant as a cooking fuel (CFPS: 63.7%; CHNS: 40.9%) than either coal or LPG (see Table 1 and Table 2). The proportion of LPG, one of the cleaner fuel types, is higher in the CFPS (25.7%) than in the CHNS (17.1%), but this outcome could result from the different periods covered by each survey (CFPS: 2010–2012 and CHNS: 1991–2009, respectively). The differences between the two datasets could also be attributed to the different geographic regions they cover. Additionally, the trends of domestic cooking fuels from 1991 to 2009 are illustrated in Figure A1. 

Two points are worth highlighting: first, the patterns of domestic cooking fuels have shifted dramatically. Specifically, wood/straw use for household cooking has declined, from 36.9% in 1991 to 17.8% in 2009. Similarly, coal use also has experienced a decrease during the same period, from 36% to 23.2%. However, the use of LPG for domestic cooking has increased significantly, from 15.2% to 22.4%. Second, although the use of wood/straw, coal has declined but LPG has increased steadily during the last decade, those three fuels are still the predominant types for household cooking fuels. Such temporal changes in our sample are very comparable to the results from Duan et al. [4] based on the data from the CHNS 1991 to 2006.

### 4.2. Cross-Sectional Evidence of HCF and Health: The CFPS and the CHNS

According to the pooled cross-sectional results for the CFPS (Table 3), women whose households rely on LPG are less likely to suffer from chronic or acute disease than those whose households rely on wood/straw (CFPS: marginal effect (ME) = −4.4%, column 1). Likewise, women in households using either coal or LPG are more likely to self-report better health status than those in households using wood/straw. 

Nevertheless, the point estimates for LPG are larger than those for coal (2.9% for LPG versus 1.9% for coal, respectively, column 2), suggesting that cleaner cooking fuels like LPG are beneficial to health. Interestingly, as for the CHNS, we also cannot observe any association between coal/LPG and chronic/acute disease (column 3). Nonetheless, we indeed find that, relative to wood/straw, LPG use is associated with better self-reported health (ME = 2.1%, column 4). To check possible effects of domestic cooking fuels on health among different age cohorts, we split the sample into two groups: women aged 16–50 and 50+. The results demonstrate that, relative to wood/straw, women aged 16–50 in households using LPG are more likely to self-report better health in the CFPS. However, this is not the case for those women aged 16–50 in the CHNS. Interestingly, we can consistently find that LPG use for cooking is associated with improved self-rated health among women aged 50+ in both the CFPS and the CHNS (see Table A3).

### 4.3. Panel Evidence for HCF and Health: The CFPS

Given the possible existence of individual unobservables or omitted factors, we also estimate random effects (ordered) probit estimates based on the CFPS data, which reveal a significantly negative association between the probability of chronic/acute diseases and cooking with LPG (ME = −5.2%, Table 4, column 1). They also reveal a significantly positive association between self-reported health and cooking with coal or LPG (ME: 2.0% for LPG versus 2.3% for coal, respectively, column 2).

Our analysis for the two age groups (15–50 and 50+) shows that, compared with wood/straw, LPG use for cooking is strongly associated with better self-reported health among women aged 50+, but not for those aged 16–50 (see Table A4).

### 4.4. Panel Evidence for HCF and Health: The CHNS

We perform a similar panel analysis with the CHNS but introduce an additional control for the time a woman spends cooking (TSC), which is divided into four categories: 1 = TSC < 1 h/day, 2 = 1 ≤ TSC < 2 h/day, 3 = 2 ≤ TSC < 3 h/day, and 4 = TSC ≥ 3 h/day, with TSC < 1 h/day as the reference. Two of the findings are worth emphasizing: First, the random effects probit estimations (Table 5, column 1) reveal no significantly negative association between domestic LPG use and chronic/acute diseases while the random effects ordered probit estimations (column 2) show a significant positive effect of LPG use on self-reported health (ME = 2.2%). Taken together, these results suggest that LPG may lead to better health outcomes. Second, none of the coefficients on time spent cooking are significant (columns 1 and 2). For the test of attrition bias, the results from random effect probit and order probit estimates indicate that this additional variable is insignificant (see Table A5). Thus, it suggests that endogenous attrition bias is not a serious issue in our case.

To detect the possible heterogeneity of domestic cooking fuel use on various different age groups, we rerun the estimates for rural women aged 15–50 and 50+ separately. The results indicate that in both age groups, relative to wood/straw, LPG use is associated with a higher probability of self-reported improved health. One interesting point to emphasize is that, for those women aged 50+, time spent cooking above 3 h/day is correlated with a lower probability of reporting better health status (see Table A6).

### 4.5. HCF and Major Risk Predictors of Cardiovascular Diseases (CVD): The CHNS

Even though China is facing a major increase in cardiovascular diseases [43,46], empirical evidence of the possible effects of household cooking fuels on CVD is quite limited [16]. High blood pressure is one of the major risk contributors for CVD [37]. Following Baumgartner et al. [8], we analyse the levels of systolic blood pressure (SBP) and diastolic blood pressure (DBP). We dichotomize the whole sample into two age groups, namely 16–50 and 50+, because the relative increased risk in cardiovascular diseases begin usually in the fifth decade of life [37]. In our analysis of the CHNS data, we note that, relative to wood/straw, cooking with coal is associated with an increase in raised levels of SBP among rural women aged 50+ (see column 2 in Table 6), but no significance is found for those women aged 16–50 and DBP among rural women aged 16+. We also introduce inflammation as another potential risk factor for CVD. Nevertheless, we find no association between domestic cooking fuels and inflammation (see column 5).

To assess the possible existence of some unobserved or omitted factors, we also estimate a fixed effects model. As Table 7 demonstrates, coal use for domestic cooking is still significantly and positively correlated with an elevated level of systolic blood pressure among women aged 50+ (see column 2), which is quite similar to that of the OLS estimates in the Table 6. Additionally, longer time exposure is also associated with raised levels of SBP among women aged 50+ (≥3 h/day) and DBP among those aged 16–50 (1–3 h/day) (columns 2 and 3). 

### 4.6. HCF and Specific Symptoms of Chronic or Acute Diseases

Finally, as Table 8 shows, we find no significant association between coal/LPG (with wood/straw as the referent) and specific disease symptoms except for significantly negative associations with palpitation in the CFPS, and with eye disease but significantly positive association with heart/chest pain in the CHNS. 

The results from the random effects probit model are illustrated in the Table 9. Two points are worth noting: First, when taking wood/straw as the reference category, we mostly fail to observe any significant association between coal/LPG and specific disease symptoms, which is consistent with the previous cross-sectional results in Table 8. Second, in the CFPS, compared with wood/straw, coal or LPG use for cooking is related to a decreased probability of fever in the CHNS (ME: −1.2% for LPG versus −0.8% for coal, respectively).

## 5. Discussion

### 5.1. Key Findings

This analysis of data from the China Family Panel Studies (CFPS) and the China Health and Nutrition Survey (CHNS) examines the association between the type of domestic cooking fuel and women’s health in rural China. Not only does it expand the extant literature by using the most recent nationally and regionally representative datasets within panel settings, it also explores how household cooking fuels are related to the health impairments associated with certain risk predictor for CVD. Our results indicate that, relative to those who use traditional biomass fuels like wood/straw, women in rural households who cook with LPG are less likely to suffer from chronic or acute diseases and more likely to report better health outcomes. It is worth noting that, this finding also holds for our age-split results, particularly for those women aged 50+, perhaps suggesting that older rural women are more likely to benefit from positive effects of cleaner fuels like LPG. Our results are well in line with those existing studies in rural China such as Zhou et al. [30] which confirm that using clean fuels like biogas (compared with biomass) is correlated with better lung function and also a decrease in risk for COPD among rural residents. Furthermore, in the CHNS, we also find that, time spent cooking is uncorrelated with health outcomes in women, which could imply that the mere use of a specific fuel may lead to HAP persistency. In other words, the spatio-temporal distribution of pollutant concentrations could be affected by house structure, room layout and ventilation [6]. However, interestingly, our results from age-split analysis indicate, for those women aged 50+, time spent cooking above 3 h/day is linked with a decreased probability of reporting better health, perhaps suggesting that older women are more likely to suffer from longer exposure to HAP. 

Our results also indicate that the use of coal for household cooking is associated with increased levels of SBP, suggesting that solid fuel use could be affecting the risk predictors for cardiovascular disease in rural China. This finding is consistent with previous epidemiological studies such as Baumgartner et al.’s [8] conclusion that indoor solid fuel combustion is related to a higher level of SBP among women aged 50+ in rural Yunnan, China. One possible explanation is that, such incomplete combustion of solid fuels like biomass and coal emits several pollutants like PM and other toxic compounds [14]. In particular, PM inhalation results in oxidative stress and systematic inflammation as well [47,48], thereby leading to elevated blood pressure levels [49]. More interestingly, we find that, relative to <1 h/day, longer time exposure is also associated with raised levels of SBP among women aged 50+ (≥3 h/day) and DBP among those aged 16–50 (1–3 h/day), which is well in line with Lee et al.’s [32] finding that longer duration of solid fuel exposure is linked with an increased risk of hypertension in the Putuo District of Shanghai. 

Regarding possible mechanisms of how different cooking fuels operate on health, exposure to biomass fuel use (like wood/straw) is associated with chronic bronchitis or COPD mainly through reduced mucociliary clearance and macrophage response [50]. In addition, exposure to biomass smoke for cooking is also a potential risk factor for lung cancer because some carcinogenic compounds are generated via polycyclic aromatic hydrocarbons [50]. Use of biomass fuel also leads to cataracts since oxidative changes happen with the absorption of toxins into the lens [50]. As Zhang and Smith [6] have highlighted, coal contains intrinsic contaminants such as sulphur, and coal burning for cooking generally takes longer than LPG or other gas combustion [51]. More importantly, the concentrations of PM and sulphur dioxide (SO_2_) are highest in the kitchen when coal is used for cooking [6], thereby giving rise to some cardiovascular diseases [8,47].

### 5.2. Limitations

In general, the advantage of our data (and main contribution of our study) is that it allows for a nationally representative analysis based on longitudinal panel data. The trade-off of using such large-scale secondary data is that it comes at the expense of more precise measures of HAP and health. Thus, certain limitations must be mentioned: One important constraint is that even though a significant proportion of rural households use a mixture of fuels [6], our dataset only permits an analysis of primary fuel use, which makes it difficult to isolate the effect of a specific fuel type. We thus cannot rule out the possibility of fuel switching/stove stacking in the household. This may also account for the beneficial but relatively weak LPG impact on possible health improvements in our analysis. Due to data limitations, we are unable to employ direct measures of the magnitude and frequency of exposure to HAP (e.g., specific amount of various toxic contaminants), although such direct proxies would be desirable. Health status and chronic/acute disease measures are self-reported and may therefore be subjected to some measurement error. Furthermore, due to data limitations, the possible effects of ventilation and type of stove (with/without chimneys) within the household cannot be analysed. Methodologically, one potential problem is the endogeneity encountered in virtually all studies on this topic [52], which in the absence of adequate instruments is difficult to solve. It is also important to highlight that, although we have examined the effects of HAP on predictors such as blood pressure and inflammation of CVD in our analysis, potential impacts of HAP on other predictors of CVD or even specific CVD are still unclear. 

### 5.3. Future Research Directions

The limitations of this study point to some interesting avenues for future research. Above all, more detailed, longitudinal and nationally representative data on HAP and health in China is needed in order to shed more light on this topic. Including direct measures of HAP in some existing large-scale health surveys in China would be interesting and most probably feasible. Although our analysis does reveal an association between cooking fuels and health, having better measures of both variables (HAP and health) and more information on the household infrastructure (e.g., ventilation, use of chimney, use of different types of fuels, exposure) would most probably render stronger effects. This, however, needs to be confirmed. Such data would also be useful in order to analyse the effects of fuel switching and stove stacking, or to better understand the association between HAP and specific health outcomes such as CVD, for which there is little evidence. Having better longitudinal data is also important as only with such data can long-term effects as well as exposure to HAP be assessed. More research is also needed on the evaluation of programmes aimed at reducing HAP in order to identify the most cost-effective approaches [52]. As pointed out by Peabody et al. [13], the Chinese Ministry of Health’s 2002 programme to improve coal stoves with chimneys [53] may not effectively solve the problem of HAP in millions of households in rural China. 

## 6. Conclusions 

Our analysis provides evidence that using cleaner fuels like LPG for domestic cooking may be associated with better health among women in rural China. Any such evidence implies that shifting from dirty fuels such as biomass to cleaner choices like LPG or electricity could improve health outcomes. This shift, however, is inherently a long-term process, one likely to be hampered by the affordability and availability of cleaner fuels in rural areas [54]. It is important to note that providing nationally representative evidence for the effects of HAP on health is quite challenging due primarily to the lack of detailed longitudinal data on both exposure to HAP and health outcomes in developing countries [55]. 

## Figures and Tables

**Table 1:** Descriptive statistics: CFPS 2010–2012.

Variable	N	Mean	Std. Dev.	Min	Max
Dependent variable					
Chronic/acute disease	12,901	0.347	0.476	0	1
Fever	12,901	0.022	0.147	0	1
Pain	12,901	0.156	0.363	0	1
Cough	12,901	0.015	0.121	0	1
Palpitation	12,901	0.033	0.177	0	1
Self-reported health (SRH)					
Poor	12,901	0.146	0.353	0	1
Fair	12,901	0.154	0.361	0	1
Good	12,901	0.171	0.376	0	1
Very good	12,901	0.262	0.440	0	1
Excellent	12,901	0.267	0.443	0	1
Household cooking fuels					
Wood/straw	12,901	0.637	0.481	0	1
Coal	12,901	0.106	0.308	0	1
liquefied petroleum gas (LPG)	12,901	0.257	0.437	0	1
Individual characteristics					
Age	12,901	46.059	16.053	16	97
Working status	12,901	0.490	0.500	0	1
Education levels					
Illiterate	12,901	0.516	0.500	0	1
Primary school	12,901	0.220	0.414	0	1
Middle school	12,901	0.194	0.395	0	1
High school	12,901	0.052	0.223	0	1
Vocational school	12,901	0.012	0.110	0	1
University or higher	12,901	0.005	0.073	0	1
Marital status	12,901	0.834	0.372	0	1
Currently smoking	12,901	0.039	0.195	0	1
Family characteristics					
Household income (log)	12,901	9.877	1.148	0.693	14.253
Household size	12,901	4.674	1.922	1	26
Drinking water	12,901	0.421	0.494	0	1
Electricity	12,901	0.944	0.230	0	1
Flushing toilet	12,901	0.209	0.407	0	1
Clean trash treatment	12,901	0.148	0.355	0	1
Community Characteristics					
Distance to the health facility (km)	12,901	1.247	1.670	0.001	9.500

Notes: Self-reported health (SRH) is measured on a 5-point-scale (1 = poor, 2 = fair, 3= good, 4 = very good and 5 = excellent). The household cooking fuels are wood/straw, coal and liquefied petroleum gas (0 = wood/straw, 1 = coal, 2 = liquefied petroleum gas (LPG)). The education level dummy is measured on a 6-point scale (1 = illiterate, 2 = primary school, 3 = middle school, 4 = high school, 5 = vocational school and 6 = university or higher). Dummies are also included for marital status (1 = married, 0 = others), working status (1 = currently employed, 0 = currently unemployed) and smoking behaviour at the time of interview (1 if the respondent had smoked for the past month, 0 otherwise).

**Table 2:** Descriptive statistics: CHNS 1991–2009.

Variable	N	Mean	Std. Dev.	Min	Max
Dependent variable					
Chronic/acute disease	15,539	0.109	0.311	0	1
Fever	15,539	0.046	0.210	0	1
Asthma	1078	0.001	0.031	0	1
Eye disease	15,539	0.004	0.066	0	1
Heart disease/chest pain	15,539	0.009	0.092	0	1
Self-reported health (SRH)					
Bad	8409	0.070	0.256	0	1
Fair	8409	0.317	0.465	0	1
Good	8409	0.492	0.500	0	1
Excellent	8409	0.121	0.326	0	1
Household cooking fuels					
Wood/straw	15,539	0.409	0.492	0	1
Coal	15,539	0.420	0.494	0	1
liquefied petroleum gas (LPG)	15,539	0.171	0.377	0	1
Individual characteristics					
Age	15539	44.959	15.728	16	97.84
Working status	15539	0.705	0.456	0	1
Education levels					
Illiterate	15,539	0.300	0.458	0	1
Primary school	15,539	0.337	0.473	0	1
Middle school	15,539	0.263	0.440	0	1
High school	15,539	0.073	0.260	0	1
Vocational school	15,539	0.020	0.140	0	1
University or higher	15,539	0.007	0.083	0	1
Marital status	15,539	0.799	0.400	0	1
Currently smoking	15,539	0.037	0.189	0	1
Time spent cooking (hours/day)	10,227	1.728	0.932	0.017	4.667
Family characteristics					
Household income (log)	15,539	9.390	1.011	1.156	13.414
Household size	15,539	4.198	1.591	1	13
Water	15,539	0.827	0.378	0	1
Flushing toilet	15,539	0.207	0.405	0	1
No excreta around the dwelling place	15,539	0.568	0.495	0	1
Electricity	15,539	0.984	0.127	0	1
Community characteristics					
Location of health facility	15,539	0.752	0.432	0	1
Distance to the health facility (km)	15,539	0.923	3.573	0	60

Notes: Self-reported health (SRH), which is only available from 1997 to 2006, is measured on a 4-point scale (1 = poor, 2 = fair, 3 = good and 4 = excellent). Asthma is only available in the year of 2009. The household cooking fuels are wood, coal and liquefied petroleum gas (0 = wood, 1 = coal, 2 = liquefied petroleum gas (LPG)). The education level dummy is measured on a 6-point scale (0 = illiterate, 1 = primary school, 2 = middle school, 3 = high school, 4 = vocational school and 5 = university or higher). Dummies are also included for marital status (1 = married, 0 = other), working status (1 = currently employed, 0 = currently unemployed) and smoking behaviour at the time of interview (1 if the respondent was currently smoking cigarettes, 0 otherwise).

**Table 3:** (Ordered) probit estimates for the effect of household cooking fuels on women’s health in rural area: CFPS 2010–2012 and CHNS 1991–2009.

Variable	CFPS		CHNS	
	(1)	(2)	(3)	(4)
	Chronic/Acute Disease	Self-Reported Health (Excellent)	Chronic/Acute Disease	Self-Reported Health (Excellent)
Coal	0.010	0.019 *	−0.008	0.009
	(0.020)	(0.011)	(0.008)	(0.007)
95% CI	[−0.030, 0.050]	[−0.002, 0.040]	[−0.025, 0.008]	[−0.005, 0.023]
LPG	−0.044 ***	0.029 ***	−0.009	0.021 ***
	(0.015)	(0.009)	(0.010)	(0.008)
95% CI	[−0.075, −0.014]	[0.012, 0.046]	[−0.028, 0.010]	[0.006, 0.037]
N	12,901	12,901	15,539	8409
Pseudo R^2^	0.065	0.147	0.090	0.081

Notes: The dependent variable is a dummy variable for whether the respondent has suffered from a chronic or acute disease (1 = yes, 0 = no) or self-reported health (SRH) measured on a 5-point scale from 1 = poor to 5 = excellent in the CFPS and a 4-point scale from 1 = bad to 4 = excellent in the CHNS. Controls include a dummy for household cooking fuel (0 = wood/straw, 1 = coal, 2 = liquefied petroleum gas (LPG), with wood/straw as the referent), individual characteristics, family characteristics, community characteristics, provincial dummies (with Liaoning as the referent in both the CFPS and CHNS) and year dummies (with 2010 and 1991 as the referents in the CFPS and CHNS, respectively). Also reported are marginal effects, which for SRH indicate the probability of excellent health. Village/neighbour or community-level clustered standard errors are in parentheses and 95% confidence intervals (CI) are in brackets. * *p* < 0.1, ** *p* < 0.05, *** *p* < 0.01.

**Table 4:** Random effects (ordered) probit estimates for the effect of household cooking fuels on women’s health in rural areas: CFPS 2010–2012.

Variable	Random Effects Probit (1) Chronic/Acute Disease	Random Effects Ordered Probit (2) Self-Reported Health (Excellent)
Coal	−0.006	0.023 **
	(0.020)	(0.010)
95% CI	[−0.046, 0.034]	[0.003, 0.044]
LPG	−0.052 ***	0.020 ***
	(0.015)	(0.007)
95% CI	[−0.081, −0.024]	[0.006, 0.035]
N	9770	10,000

Notes: The dependent variable is either a dummy for whether the respondent has suffered from a chronic or acute illness (1 = yes, 0 = no) or a 5-point measure of self-reported health (SRH, from 1 = poor to 5 = excellent). The controls are domestic cooking fuel (0 = wood/straw, 1 = coal, 2 = liquefied petroleum gas (LPG), with wood/straw as the referent), age, age squared, household income and household size. Also reported are marginal effects, which for SRH indicate the probability of excellent health. Standard errors are in parentheses and 95% confidence intervals (CI) are in brackets. * *p* < 0.1, ** *p* < 0.05, *** *p* < 0.01.

**Table 5:** Random effects (ordered) probit estimates for the effect of household cooking fuels on women’s health in rural areas: CHNS 1991–2009.

Variable	Random Effects Probit (1) Chronic/Acute Disease	Random Effects Ordered Probit (2) Self-Reported Health (Excellent)
Coal	−0.007	0.002
	(0.006)	(0.005)
95% CI	[−0.019, 0.005]	[−0.008, 0.012]
LPG	−0.013	0.022 ***
	(0.008)	(0.006)
95% CI	[−0.030, 0.004]	[0.009, 0.034]
1 ≤ TSC < 2 h/day	−0.003	−0.005
	(0.008)	(0.006)
95% CI	[−0.018, 0.011]	[−0.016, 0.006]
2 ≤ TSC < 3 h/day	−0.011	0.003
	(0.008)	(0.006)
95% CI	[−0.027, 0.005]	[−0.010, 0.015]
TSC ≥ 3 h/day	−0.012	−0.011
	(0.009)	(0.008)
95% CI	[−0.029, 0.006]	[−0.026, 0.004]
N	10,090	7023

Notes: The dependent variable is either a dummy for whether or not the respondent has suffered from a chronic or acute disease or a 4-point measure of self-reported health (1 = bad, 2 = fair, 3 = good and 4 = excellent). The controls are cooking fuel (0 = wood, 1 = coal, 2 = liquefied petroleum gas (LPG), with wood/straw as the referent), a dummy for time spent cooking (TSC, 1 = TSC < 1 h/day, 2 = 1 ≤ TSC < 2 h/day, 3 = 2 ≤ TSC < 3 h/day, 4 = TSC ≥ 3 h/day, with group 1 as the referent), age, age squared, household income (inflated to 2011) and household size. Also reported are marginal effects, which for SRH indicate the probability of excellent health. Standard errors are in parentheses and 95% confidence intervals (CI) are in brackets. * *p* < 0.1, ** *p* < 0.05, *** *p* < 0.01.

**Table 6:** OLS/probit estimates for the effect of household cooking fuels on women’s blood pressure/inflammation in rural areas: CHNS.

Variable	OLS Estimate		Probit Estimate		
	16–50	50+	16–50	50+	
	Systolic Blood Pressure		Diastolic Blood Pressure		Inflammation
	(1)	(2)	(3)	(4)	(5)
Coal	0.110	2.295 *	0.567	0.957	0.025
	(0.703)	(1.282)	(0.453)	(0.760)	(0.032)
95% CI	[−1.279, 1.499]	[−0.239, 4.829]	[−0.329, 1.462]	[−0.545, 2.459]	[−0.037, 0.088]
LPG	0.345	0.936	0.512	0.873	0.021
	(0.750)	(1.427)	(0.480)	(0.841)	(0.032)
95% CI	[−1.138, 1.828]	[−1.883, 3.755]	[−0.437, 1.460]	[−0.789, 2.535]	[−0.041, 0.083]
N	6389	3838	6389	3838	1637
Pseudo/Adjusted R^2^	0.146	0.109	0.128	0.062	0.058

Notes: The dependent variable is a dummy for whether the respondent has suffered from inflammation (1 = yes, 0 = no) or from levels of systolic and diastolic blood pressure. The information on blood pressure is available from 1991 to 2009; that on inflammation, only in 2009. The controls include a dummy for household cooking fuel (0 = wood, 1 = coal, 2 = liquefied petroleum gas (LPG), with wood as the referent), a dummy for time spent cooking (TSC, 1 = TSC < 1 h/day, 2 = 1 ≤ TSC < 2 h/day, 3 = 2 ≤ TSC < 3 h/day, 4 = TSC ≥ 3 h/day, with group 1 as the referent), individual characteristics, family characteristics, community characteristics, provincial dummies (with Liaoning as the referent) and year dummies (with 1991 as the referent). Marginal effects are reported for the probit estimate of inflammation. Community-level clustered standard errors are in parentheses and 95% confidence intervals (CI) are in brackets. * *p* < 0.1, ** *p* < 0.05, *** *p* < 0.01.

**Table 7:** Fixed effects estimates for the effect of household cooking fuels on women’ blood pressure in rural area: CHNS 1991–2009.

Variable	Systolic Blood Pressure		Diastolic Blood Pressure	
	(1)	(2)	(3)	(4)
	16–50	50+	16–50	50+
Coal	−0.599	2.267 *	0.288	0.079
	(0.669)	(1.229)	(0.481)	(0.707)
95% CI	[−1.910, 0.713]	[−0.144, 4.677]	[−0.655, 1.232]	[−1.308, 1.466]
LPG	−0.131	−0.573	0.654	−0.215
	(0.768)	(1.438)	(0.553)	(0.827)
95% CI	[−1.637, 1.375]	[−3.392, 2.246]	[−0.430, 1.738]	[−1.838, 1.407]
1 ≤ TSC < 2 h/day	0.524	1.536	1.076 **	−0.111
	(0.612)	(1.006)	(0.440)	(0.579)
95% CI	[−0.676, 1.724]	[−0.435, 3.508]	[0.212, 1.940]	[−1.246, 1.023]
2 ≤ TSC < 3 h/day	−0.036	1.731	0.920 *	−0.110
	(0.664)	(1.129)	(0.478)	(0.649)
95% CI	[−1.337, 1.265]	[−0.483, 3.944]	[−0.016, 1.857]	[−1.384, 1.164]
TSC ≥ 3 h/day	−1.064	2.603 **	−0.008	0.417
	(0.726)	(1.300)	(0.523)	(0.748)
95% CI	[−2.488, 0.359]	[0.055, 5.152]	[−1.033, 1.017]	[−1.049, 1.884]
N	6252	3838	6252	3838
R^2^	0.088	0.102	0.066	0.028

Notes: The dependent variables are levels of systolic/diastolic blood pressure. Controls are cooking fuel choices (0 = wood, 1 = coal, 2 = liquefied petroleum gas (LPG), wood/straw as the reference), a dummy for time spent cooking (TSC, 1 = TSC < 1 hour/day, 2 = 1 ≤ TSC < 2 h/day, 3 = 2 ≤ TSC < 3 h/day, 4 = TSC ≥ 3 h/day, with group 1 as the referent), age, age squared, household income (inflated to 2011) and household size. Standard errors are in parentheses and 95% confidence intervals (CI) are in brackets. * *p* < 0.1, ** *p* < 0.05, *** *p* < 0.01.

**Table 8:** Probit estimates for the effect of domestic cooking fuels on women’s health (specific symptoms) in rural areas: CFPS 2010–2012 & CHNS 1991–2009.

**CFPS**				
**Variable**	**Fever**	**Cough**	**Pain**	**Palpitation**
Coal	−0.003	0.003	0.007	0.006
	(0.005)	(0.004)	(0.015)	(0.005)
95% CI	[−0.013, 0.007]	[−0.005, 0.010]	[−0.021, 0.036]	[−0.003, 0.016]
LPG	0.001	0.003	−0.018	−0.008 *
	(0.004)	(0.003)	(0.012)	(0.004)
95% CI	[−0.006, 0.008]	[−0.003, 0.009]	[−0.041, 0.005]	[−0.017, 0.0003]
N	12,637	12,623	12,901	12,901
Pseudo R^2^	0.023	0.031	0.059	0.072
**CHNS**				
**Variable**	**Fever/Cough**	**Asthma**	**Eye**	**Heart/Chest Pain**
Coal	−0.003	−0.007	−0.003 *	0.004 *
	(0.005)	(0.007)	(0.002)	(0.002)
95% CI	[−0.013, 0.007]	[−0.021, 0.006]	[−0.006, 0.0002]	[−0.0004, 0.008]
LPG	−0.001	−0.007	−0.004 **	0.003
	(0.006)	(0.010)	(0.002)	(0.002)
95% CI	[−0.012, 0.010]	[−0.027, 0.013]	[−0.008, −0.001]	[−0.002, 0.008]
N	15,539	1078	14,299	15,539
Pseudo R^2^	0.081	0.297	0.208	0.172

Notes: The dependent variable is a dummy for whether the respondent has suffered from fever, cough, asthma, pain, palpitation, eye or heart disease/chest pain (1 = yes, 0 = no). Asthma is only available in the year of 2009 for the CHNS. The controls are a dummy for household cooking fuel (0 = wood/straw, 1 = coal, 2 = liquefied petroleum gas (LPG), with wood/straw as the referent), individual characteristics, family characteristics, community characteristics, provincial dummies (with Liaoning as the referent in both the CFPS and CHNS) and year dummies (with 2010 and 1991 as the referent in the CFPS and CHNS, respectively). For the CHNS, controls are similar to those in the CFPS but add dummies for time spent cooking (TSC, 1 = TSC < 1 h/day, 2 = 1 ≤ TSC < 2 h/day, 3 = 2 ≤ TSC < 3 h/day, 4 = TSC ≥ 3 h/day, with group 1 as the referent). Marginal effects are reported. Village/neighbour or community-level clustered standard errors are in parentheses and 95% confidence intervals (CI) are in brackets.* *p* < 0.1, ** *p* < 0.05, *** *p* < 0.01.

**Table 9:** Random effects probit estimates for the effect of domestic cooking fuels on women’s health (specific symptoms) in rural areas: CFPS 2010–2012 & CHNS 1991–2009.

**CFPS**				
**Variable**	**Fever**	**Cough**	**Pain**	**Palpitation**
Coal	−0.002	0.003	0.001	0.003
	(0.004)	(0.002)	(0.001)	(0.003)
95% CI	[−0.010, 0.007]	[−0.002, 0.007]	[−0.002, 0.004]	[−0.002, 0.008]
LPG	0.002	−0.002	0.002	−0.003
	(0.003)	(0.002)	(0.001)	(0.002)
95% CI	[−0.004, 0.007]	[−0.006, 0.001]	[−0.001, 0.004]	[−0.007, 0.001]
N	10,002	10,002	10,002	10,002
**CHNS**				
**Variable**	**Fever**	**Eye**	**Heart/Chest Pain**	
Coal	−0.008 *	−0.001	0.001	
	(0.004)	(0.001)	(0.001)	
95% CI	[−0.017, 0.00004]	[−0.003, 0.001]	[−0.001, 0.003]	
LPG	−0.012 **	−0.001	0.001	
	(0.006)	(0.001)	(0.001)	
95% CI	[−0.024, −0.0003]	[−0.003, 0.001]	[−0.002, 0.003]	
N	10,090	10,090	10,090	

Notes: The dependent variable is a dummy for whether the respondent has suffered from fever, cough, pain, palpitation, eye or heart disease/chest pain (1 = yes, 0 = no). In the CFPS, the controls are a dummy for household cooking fuel (0 = wood/straw, 1 = coal, 2 = liquefied petroleum gas (LPG), with wood/straw as the referent), age, age squared, household income (inflated to 2012) and household size. For the CHNS, controls are similar to those in the CFPS but add dummies for time spent cooking (TSC, 1 = TSC < 1 h/day, 2 = 1 ≤ TSC < 2 h/day, 3 = 2 ≤ TSC < 3 h/day, 4 = TSC ≥ 3 h/day, with group 1 as the referent). Marginal effects are reported. Standard errors are in parentheses and 95% confidence intervals (CI) are in brackets.* *p* < 0.1, ** *p* < 0.05, *** *p* < 0.01.

## Appendix A

**Table A1:** Definition of independent variables.

Independent Variables	Definitions
Individual characteristics	
Age	Years of age
Working status	1, if the respondent is currently employed and 0 otherwise.
Education levels	Measured on a 6-point scale recoded as a dummy variable: 1 = illiterate, 2 = primary school, 3 = middle school, 4 = high school, 5 = vocational school and 6 = university or higher.
Marital status	1, if the respondent is married/living together with a partner and 0 if the respondent is divorced/separated/widowed.
Currently smoking	1, if the respondent has smoked in the past month and 0 otherwise.
Time spent cooking	In the CHNS, based on the interview question: “During the previous week, how much time (in hours) did you spend per day, on average, cooking food for the household?”.
Family characteristics	
Household cooking fuels	Measured on a 3-point scale, 0 = wood/straw, 1 = coal and 2 = liquefied petroleum gas (LPG), based on the interview question: “What kind of fuel does you household normally use for cooking?”.
Household income	Total amount of household income (in Yuan, adjusted to 2012 in the CFPS and to 2011 in the CHNS).
Household size	Number of people in the household.
Drinking water	In the CFPS, 1 if the household’s drinking source is tap water or mineral/purified water, 0 otherwise. In the CHNS, 1 if the household’s water source is a water plant or ground water above 5 m deep, 0 otherwise.
Electricity	In the CFPS, 1 if the household has occasional or no power outage, 0 otherwise. In the CHNS, 1 if electric facilities are accessible for the household, 0 otherwise.
Flushing toilet	In the CFPS, 1 if the household mostly use an indoor/outdoor flushing toilet, 0 otherwise. In the CHNS, 1 if the household can access an in-house/out-house flushing toilet facility, 0 otherwise.
Clean trash treatment	In the CFPS, 1 if the household dumps the trash in the public dustbin/garbage can, 0 otherwise.
Existence of excreta	In the CHNS, 1 if there is no excreta around the dwelling place, 0 otherwise.
Community Characteristics	
Location of ealth facility	In the CHNS, the availability of health facilities in the community is defined by a dummy variable equal to 1 if a health facility is located in the village/neighbourhood and 0 if in another village/town/city or in the respondent’s city but in a different neighbourhood.
Distance to the health facility	Distance (in km) in the community to the nearest health facility like hospital/medical center.

**Table A2:** Summary of health measures, data and methods.

Health Measures	Description	Definition	Data Source	Years	Methodology
Self-reported acute/chronic disease	Have you felt any physical discomfort during the preceding two weeks?	A binary variable equal to 1 if the respondent has felt discomfort, and 0 otherwise.	CFPS	2010–2012	Probit model Random effect probit model
	Have you suffered from a chronic or acute disease during the past 4 weeks?	A dummy that equals 1 if the respondent has suffered from a chronic or acute disease, and 0 otherwise.	CHNS	1991–2009	Probit model Random effect probit model
Self-reported health (SRH)	How would you rate your health status? 1 = excellent, 2 = very good, 3 = good, 4 = fair and 5 = poor.	A 5-point scale ranging from 1 = poor to 5 = excellent.	CFPS	2010–2012	Ordered probit model Random effect ordered probit model
	Right now, how would you describe your health compared to that of other people your age? 1 = bad; 2 = fair; 3 = good; 4 = excellent.	A 4-point scale ranging from 1 = bad to 4 = excellent.	CHNS	1997–2006	Ordered probit model Random effect ordered probit model
Systolic blood pressure (SBP)	Measurements are taken three times by a health professional using a mercury sphygmomanometer.	The average value of SBP based on the second and third measurements.	CHNS	1991–2009	Ordinary least squares model Fixed effect model
Diastolic blood pressure (DBP)	Measurements are taken three times by a health professional using a mercury sphygmomanometer.	The average value of SBP based on the second and third measurements.	CHNS	1991–2009	Ordinary least square model Fixed effect model
Inflammation	Using high sensitivity C-reactive protein.	A dummy equal to 1 if the high sensitivity C-reactive protein exceeds 3 mg/dL, 0 otherwise.	CHNS	2009	Probit model

**Table A3:** Ordered probit estimates for cooking fuel choice on women self-reported health in rural area: CFPS 2010–2012 and CHNS 1991–2009.

Variable	CFPS		CHNS	
	(1)	(2)	(3)	(4)
	16–50	50+	16–50	50+
Coal	0.010	0.027 **	0.016	0.003
	(0.015)	(0.012)	(0.012)	(0.006)
95% CI	[−0.020, 0.039]	[0.004, 0.050]	[−0.008, 0.041]	[−0.009, 0.015]
LPG	0.025 **	0.035 ***	0.014	0.022 ***
	(0.012)	(0.011)	(0.013)	(0.007)
95% CI	[0.002, 0.048]	[0.014, 0.056]	[−0.011, 0.038]	[0.008, 0.037]
N	7958	4945	5107	3302
Pseudo R^2^	0.140	0.109	0.052	0.042

Notes: The dependent variable is self-reported health (SRH) measured on a 5-point scale from 1 = poor to 5 = excellent in the CFPS or a 4-point scale from 1 = poor to 4 = excellent in the CHNS. Controls include dummies of household cooking fuel choices (0 = wood, 1 = coal, 2 = liquefied petroleum gas (LPG), wood as the reference), individual characteristics (including age, age squared, education level, marital status, job status, current smoking behavior, participation into food preparation and cooking), family characteristics (translog household income inflated to 2011, household size, the availability of safe drinking water, sanitation and clean trash treatment), community characteristics (distance to the health facility), provincial dummies (Liaoning as the reference) and year dummies (2010 as the reference). Also reported are marginal effects, which for SRH indicate the probability of excellent health. Village/neighbour clustered standard errors are in parentheses and 95% confidence intervals (CI) are in brackets. * *p* < 0.1, ** *p* < 0.05, *** *p* < 0.01.

**Table A4:** Random effects ordered probit estimates for cooking fuel choice on women’s self-reported health in rural area: CFPS 2010–2012.

Variable	Random Effects Ordered Probit: 16–50	Random Effects Ordered Probit: 50+
Coal	0.021	0.024 *
	(0.016)	(0.013)
95% CI	[−0.011, 0.052]	[−0.001, 0.048]
LPG	0.012	0.027 ***
	(0.011)	(0.009)
95% CI	[−0.010, 0.033]	[0.009, 0.046]
N	6011	3989

Notes: The dependent variable is self-reported health (SRH) measured on a 5-point-scale variable of self-reported health status (from 1 = very unhealthy to 5 = very healthy). Controls include cooking fuel choices (0 = wood, 1 = coal, 2 = liquefied petroleum gas (LPG), wood as the reference), age, age squared, household income (inflated to 2011) and household size. Also reported are marginal effects, which for SRH indicate the probability of excellent health. Standard errors are in parentheses and 95% confidence intervals (CI) are in brackets. * *p* < 0.1, ** *p* < 0.05, *** *p* < 0.01.

**Table A5:** Random effects (ordered) probit estimates for the effect of household cooking fuels on women’s health in rural areas: CHNS 1991–2009 (the test of attrition bias).

Variable	Chronic/Acute Disease	Self-Reported Health (Excellent)
	Random Effects Probit	Random Effects Ordered Probit
Coal	−0.007	0.002
	(0.006)	(0.005)
95% CI	[−0.019, 0.005]	[−0.008, 0.012]
LPG	−0.013	0.022 ***
	(0.009)	(0.006)
95% CI	[−0.029, 0.004]	[0.009, 0.034]
1 ≤ TSC < 2 h/day	−0.003	−0.005
	(0.008)	(0.006)
95% CI	[−0.018, 0.012]	[−0.016, 0.006]
2 ≤ TSC < 3 h/day	−0.011	0.003
	(0.008)	(0.006)
95% CI	[−0.027, 0.005]	[−0.010, 0.015]
TSC ≥ 3 h/day	−0.012	−0.011
	(0.009)	(0.008)
95% CI	[−0.029, 0.006]	[−0.026, 0.004]
Number of surveyed years	0.001	−0.0005
	(0.002)	(0.002)
95% CI	[−0.003, 0.005]	[−0.004, 0.003]
N	10,090	7023

Notes: The dependent variable is a dummy variable of whether the respondent has suffered from a chronic or acute disease/a 4-point-scale measure of self-reported health (1 = bad, 2 = fair, 3 = good and 4 = excellent). The controls are cooking fuel (0 = wood, 1 = coal, 2 = liquefied petroleum gas (LPG), with wood/straw as the referent), a dummy for time spent cooking (TSC, 1 = TSC < 1 h/day, 2 = 1 ≤ TSC < 2 h/day, 3 = 2 ≤ TSC < 3 h/day, 4 = TSC ≥ 3 h/day, with group 1 as the referent), household income (inflated to 2011), household size and the number of surveyed years. Also reported are marginal effects, which for SRH indicate the probability of excellent health. Standard errors are in parentheses and 95% confidence intervals (CI) are in brackets. * *p* < 0.1, ** *p* < 0.05, *** *p* < 0.01.

**Table A6:** Random effects ordered probit estimates for household cooking fuels on women self-reported health in rural area: CHNS 1991–2009.

Variable	Random Effects Ordered Probit: 16–50	Random Effects Ordered Probit: 50+
Coal	−0.001	0.005
	(0.009)	(0.004)
95% CI	[−0.019, 0.017]	[−0.004, 0.013]
LPG	0.020 *	0.020 ***
	(0.011)	(0.006)
95% CI	[−0.002, 0.041]	[0.008, 0.031]
1 ≤ TSC < 2 h/day	0.001	−0.007
	(0.010)	(0.005)
95% CI	[−0.019, 0.020]	[−0.016, 0.003]
2 ≤ TSC < 3 h/day	0.007	−0.001
	(0.011)	(0.005)
95% CI	[−0.015, 0.029]	[−0.012, 0.009]
TSC ≥ 3 h/day	−0.001	−0.014 **
	(0.013)	(0.007)
95% CI	[−0.027, 0.026]	[−0.028, −0.001]
N	4085	2938

Notes: The dependent variable is self-reported health (SRH) measured on a 4-point scale from 1 = bad to 4 = excellent. Controls include cooking fuel choices (0 = wood, 1 = coal, 2 = liquefied petroleum gas (LPG), wood/straw as the reference), dummies of time spent cooking (TSC, 1 = TSC < 1 h/day, 2 = 1 ≤ TSC < 2 h/day, 3 = 2 ≤ TSC < 3 h/day, 4 = TSC ≥ 3 h/day, with group 1 as the referent), age, age squared, household income (inflated to 2011) and household size. Also reported are marginal effects, which for SRH indicate the probability of excellent health. Standard errors are in parentheses and 95% confidence intervals (CI) are in brackets. * *p* < 0.1, ** *p* < 0.05, *** *p* < 0.01.
